# Bis(dimethyl sulfoxide)­hydridobis(triphenyl­phosphane)cobalt(I)

**DOI:** 10.1107/S1600536810029466

**Published:** 2010-07-31

**Authors:** Marko Hapke, Nico Weding, Anke Spannenberg

**Affiliations:** a Leibniz-Institut für Katalyse e. V. an der Universität Rostock, Albert-Einstein-Strasse 29a, 18059 Rostock, Germany

## Abstract

The title compound, [CoH(C_18_H_15_P)_2_(C_2_H_6_OS)_2_], was synthesized by the reaction of chloridotris(triphenyl­phosphane)cobalt(I), [ClCo(PPh_3_)_3_], in the presence of one equivalent potassium hydridotris(pyrazol­yl)borate in dimethyl sulfoxide. The structure displays a distorted trigonal-pyramidally coordinated cobalt(I) atom, with two phosphane ligands and one DMSO ligand in the equatorial plane. The coordination is completed by one further DMSO ligand and the anionic hydride in the axial positions.

## Related literature

For the hydro­formyl­ation of alkenes, see: Roelen (1938 ▶). Derivatives of the title compound, starting from Co_2_(CO)_8_, have been synthesized by reaction with hydrogen, see: Hieber & Leutert (1931 ▶). A related compound, [HCo(P(OEt)_3_)_4_], obtained by reaction of cobalt halides and sodium borohydride has been reported by Kruse & Atalla (1968 ▶). Its mol­ecular structure in the crystal was determined by Choi & Park (2003 ▶).
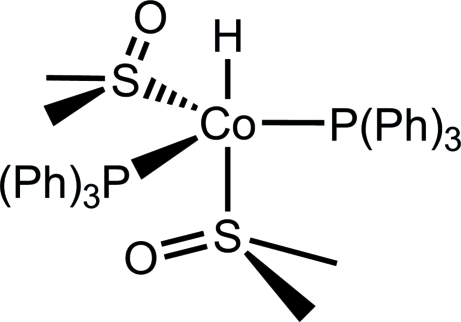


## Experimental

### 

#### Crystal data

[CoH(C_18_H_15_P)_2_(C_2_H_6_OS)_2_]
*M* *_r_* = 740.73
Monoclinic, 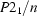

*a* = 10.5625 (2) Å
*b* = 21.4211 (3) Å
*c* = 15.8427 (4) Å
β = 93.4988 (18)°
*V* = 3577.88 (13) Å^3^
*Z* = 4
Mo *K*α radiation
μ = 0.72 mm^−1^
*T* = 200 K
0.50 × 0.27 × 0.12 mm

#### Data collection

Stoe IPDS II diffractometer
Absorption correction: numerical (*X-SHAPE* and *X-RED32*; Stoe & Cie, 2005 ▶) *T*_min_ = 0.786, *T*_max_ = 0.918
59344 measured reflections
8221 independent reflections
6773 reflections with *I* > 2σ(*I*)
*R*_int_ = 0.032

#### Refinement

*R*[*F* ^2^ > 2σ(*F* ^2^)] = 0.026
*wR*(*F* ^2^) = 0.070
*S* = 0.96
8221 reflections
432 parameters
3 restraints
H atoms treated by a mixture of independent and constrained refinement
Δρ_max_ = 0.37 e Å^−3^
Δρ_min_ = −0.25 e Å^−3^

### 

Data collection: *X-AREA* (Stoe & Cie, 2005 ▶); cell refinement: *X-AREA*; data reduction: *X-AREA*; program(s) used to solve structure: *SHELXS97* (Sheldrick, 2008 ▶); program(s) used to refine structure: *SHELXL97* (Sheldrick, 2008 ▶); molecular graphics: *XP* in *SHELXTL* (Sheldrick, 2008 ▶); software used to prepare material for publication: *SHELXTL*.

## Supplementary Material

Crystal structure: contains datablocks I, global. DOI: 10.1107/S1600536810029466/bt5307sup1.cif

Structure factors: contains datablocks I. DOI: 10.1107/S1600536810029466/bt5307Isup2.hkl

Additional supplementary materials:  crystallographic information; 3D view; checkCIF report

## Figures and Tables

**Fig. 1.**
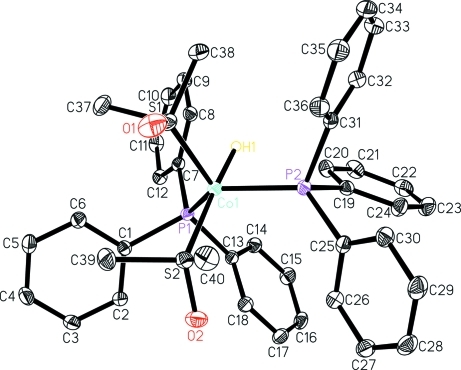
The molecular structure of the title compound showing the atom-labelling scheme. Displacement ellipsoids are drawn at the 30% probability level. Hydrogen atoms are omitted for clarity.

## supplementary crystallographic information

### Comment

The hydroformylation of alkenes was discovered by Roelen (1938). In the hydroformylation reaction, alkenes react with carbon monoxide and hydrogen, in the presence of a transition metal catalyst, to form aldehydes containing an additional carbon atom. The first generation of catalysts was formed *in situ* by the use of Co_2_(CO)_8_ with molecular hydrogen yielding [HCo(CO)_4_]. The loss of one carbonyl produces the active species [HCo(CO)_3_], which can enter the catalytic cycle. Since than cobalt hydrides have been in the focus and interest for many synthetic applications. Herein we report the hydrido-bis(triphenylphosphane)-bis(dimethylsulfoxide)cobalt(I) complex which was prepared comparable to an earlier report from Kruse *et al.* (1968).

The molecular structure of the title compound displays a distorted trigonal bipyramidal coordination geometry at the cobalt(I) centre with two phosphane ligands and one DMSO ligand in the equatorial plane. The cobalt centre lies 0.376 Å out of the P1,P2,S1 plane. The P—Co—P angle consist of 118.73 (2) °. The coordination geometry is completed by one further DMSO ligand and the hydride.

### Experimental

Chloro-tris(triphenylphosphane)cobalt(I) (0.7 g, 0.79 mmol) and potassium hydro-tris(pyrazolyl)borate (0.2 g, 0.79 mmol) were weighted into a Schlenk flask in the glove-box. The reaction flask was connected to a Schlenk line outside the box and 20 ml of dimethylsulfoxide were added. Stirring at room temperature for 20 h resulted in a deep red solution. Extraction with pentane (2 *x* 15 ml) and concentration of the dimethylsulfoxide phase led to precipitation of red crystals of the title compound (yield < 5%).

### Refinement

The H atom bonded to Co was found from difference Fourier map and refined freely. All other H atoms were placed in idealized positions with d(C—H) = 0.98 (CH_3_) and 0.95 Å (CH) and refined using a riding model with *U*_iso_(H) fixed at 1.5 *U*_eq_(C) for CH_3_ and 1.2 *U*_eq_(C) for CH. The distances C2-C3 and C3-C4, C20-C21 and C21-C22, C32-C33 and C33-C34 were restrained to be equal within an effective e.s.d. of 0.002Å.

### Crystal data

**Table:**

[CoH(C_18_H_15_P)_2_(C_2_H_6_OS)_2_]	*F*(000) = 1552
*M**_r_* = 740.73	*D*_x_ = 1.375 Mg m^−^^3^
Monoclinic, *P*2_1_/*n*	Mo *K*α radiation, λ = 0.71073 Å
Hall symbol: -P 2yn	Cell parameters from 11909 reflections
*a* = 10.5625 (2) Å	θ = 1.6–28.4°
*b* = 21.4211 (3) Å	µ = 0.72 mm^−^^1^
*c* = 15.8427 (4) Å	*T* = 200 K
β = 93.4988 (18)°	Prism, orange
*V* = 3577.88 (13) Å^3^	0.50 × 0.27 × 0.12 mm
*Z* = 4	

### Data collection

**Table:**

Stoe IPDS II diffractometer	8221 independent reflections
Radiation source: fine-focus sealed tube	6773 reflections with *I* > 2σ(*I*)
graphite	*R*_int_ = 0.032
ω scans	θ_max_ = 27.5°, θ_min_ = 1.6°
Absorption correction: numerical (*X-SHAPE* and *X-RED32*; Stoe & Cie, 2005)	*h* = −13→13
*T*_min_ = 0.786, *T*_max_ = 0.918	*k* = −27→26
59344 measured reflections	*l* = −20→20

### Refinement

**Table:**

Refinement on *F*^2^	Primary atom site location: structure-invariant direct methods
Least-squares matrix: full	Secondary atom site location: difference Fourier map
*R*[*F*^2^ > 2σ(*F*^2^)] = 0.026	Hydrogen site location: inferred from neighbouring sites
*wR*(*F*^2^) = 0.070	H atoms treated by a mixture of independent and constrained refinement
*S* = 0.96	*w* = 1/[σ^2^(*F*_o_^2^) + (0.0493*P*)^2^] where *P* = (*F*_o_^2^ + 2*F*_c_^2^)/3
8221 reflections	(Δ/σ)_max_ = 0.001
432 parameters	Δρ_max_ = 0.37 e Å^−^^3^
3 restraints	Δρ_min_ = −0.24 e Å^−^^3^

### Special details

**Table:**

Geometry. All e.s.d.'s (except the e.s.d. in the dihedral angle between two l.s. planes) are estimated using the full covariance matrix. The cell e.s.d.'s are taken into account individually in the estimation of e.s.d.'s in distances, angles and torsion angles; correlations between e.s.d.'s in cell parameters are only used when they are defined by crystal symmetry. An approximate (isotropic) treatment of cell e.s.d.'s is used for estimating e.s.d.'s involving l.s. planes.
Refinement. Refinement of *F*^2^ against ALL reflections. The weighted *R*-factor *wR* and goodness of fit *S* are based on *F*^2^, conventional *R*-factors *R* are based on *F*, with *F* set to zero for negative *F*^2^. The threshold expression of *F*^2^ > σ(*F*^2^) is used only for calculating *R*-factors(gt) *etc*. and is not relevant to the choice of reflections for refinement. *R*-factors based on *F*^2^ are statistically about twice as large as those based on *F*, and *R*- factors based on ALL data will be even larger.

### Fractional atomic coordinates and isotropic or equivalent isotropic displacement parameters (Å^2^)

**Table:**

	*x*	*y*	*z*	*U*_iso_*/*U*_eq_	
C1	0.84405 (12)	0.29130 (6)	0.07852 (8)	0.0229 (3)	
C2	0.84028 (15)	0.28531 (7)	−0.00879 (9)	0.0304 (3)	
H2A	0.8703	0.2481	−0.0334	0.036*	
C3	0.79306 (18)	0.33323 (6)	−0.06027 (10)	0.0415 (4)	
H3A	0.7904	0.3285	−0.1200	0.050*	
C4	0.74978 (17)	0.38784 (7)	−0.02578 (11)	0.0401 (4)	
H4A	0.7141	0.4198	−0.0614	0.048*	
C5	0.75878 (17)	0.39566 (8)	0.06092 (11)	0.0382 (4)	
H5A	0.7324	0.4337	0.0852	0.046*	
C6	0.80645 (16)	0.34784 (7)	0.11226 (10)	0.0328 (3)	
H6A	0.8137	0.3537	0.1718	0.039*	
C7	1.02598 (12)	0.26634 (6)	0.21005 (8)	0.0222 (3)	
C8	1.04236 (13)	0.26494 (7)	0.29762 (9)	0.0258 (3)	
H8A	0.9809	0.2445	0.3292	0.031*	
C9	1.14670 (15)	0.29276 (7)	0.33999 (10)	0.0316 (3)	
H9A	1.1553	0.2921	0.4000	0.038*	
C10	1.23767 (15)	0.32124 (7)	0.29451 (11)	0.0348 (3)	
H10A	1.3111	0.3387	0.3230	0.042*	
C11	1.22190 (14)	0.32440 (7)	0.20740 (11)	0.0324 (3)	
H11A	1.2838	0.3448	0.1762	0.039*	
C12	1.11604 (14)	0.29798 (7)	0.16529 (9)	0.0271 (3)	
H12A	1.1047	0.3014	0.1055	0.033*	
C13	0.96617 (13)	0.17149 (6)	0.08568 (8)	0.0228 (3)	
C14	1.09483 (14)	0.15752 (7)	0.09201 (9)	0.0289 (3)	
H14A	1.1501	0.1804	0.1301	0.035*	
C15	1.14359 (16)	0.11037 (8)	0.04310 (10)	0.0349 (3)	
H15A	1.2316	0.1011	0.0485	0.042*	
C16	1.06507 (16)	0.07706 (7)	−0.01308 (10)	0.0336 (3)	
H16A	1.0988	0.0451	−0.0467	0.040*	
C17	0.93693 (16)	0.09046 (7)	−0.02010 (10)	0.0327 (3)	
H17A	0.8824	0.0678	−0.0590	0.039*	
C18	0.88760 (14)	0.13677 (7)	0.02925 (9)	0.0270 (3)	
H18A	0.7991	0.1451	0.0247	0.032*	
C19	0.91922 (13)	0.05062 (7)	0.25711 (8)	0.0249 (3)	
C20	1.03066 (13)	0.08500 (7)	0.26426 (10)	0.0303 (3)	
H20A	1.0268	0.1289	0.2727	0.036*	
C21	1.14762 (14)	0.05622 (7)	0.25922 (10)	0.0365 (4)	
H21A	1.2230	0.0805	0.2642	0.044*	
C22	1.15513 (16)	−0.00756 (8)	0.24693 (10)	0.0382 (4)	
H22A	1.2351	−0.0272	0.2425	0.046*	
C23	1.04497 (17)	−0.04240 (8)	0.24121 (11)	0.0405 (4)	
H23A	1.0492	−0.0863	0.2337	0.049*	
C24	0.92830 (15)	−0.01364 (7)	0.24641 (10)	0.0336 (3)	
H24A	0.8533	−0.0382	0.2426	0.040*	
C25	0.66405 (13)	0.02838 (7)	0.21015 (9)	0.0254 (3)	
C26	0.67228 (14)	0.02436 (7)	0.12283 (9)	0.0295 (3)	
H26A	0.7227	0.0536	0.0948	0.035*	
C27	0.60789 (15)	−0.02173 (8)	0.07636 (10)	0.0344 (3)	
H27A	0.6162	−0.0245	0.0171	0.041*	
C28	0.53165 (16)	−0.06371 (8)	0.11571 (11)	0.0391 (4)	
H28A	0.4861	−0.0948	0.0836	0.047*	
C29	0.52226 (16)	−0.06019 (8)	0.20204 (11)	0.0385 (4)	
H29A	0.4700	−0.0890	0.2294	0.046*	
C30	0.58849 (14)	−0.01489 (7)	0.24947 (10)	0.0313 (3)	
H30A	0.5822	−0.0134	0.3090	0.038*	
C31	0.73647 (14)	0.07445 (6)	0.37623 (9)	0.0254 (3)	
C32	0.83543 (15)	0.06171 (7)	0.43584 (9)	0.0316 (3)	
H32A	0.9193	0.0574	0.4181	0.038*	
C33	0.81317 (16)	0.05521 (8)	0.52067 (9)	0.0395 (4)	
H33A	0.8819	0.0474	0.5608	0.047*	
C34	0.69134 (18)	0.06003 (8)	0.54708 (10)	0.0419 (4)	
H34A	0.6757	0.0542	0.6050	0.050*	
C35	0.59222 (18)	0.07343 (8)	0.48892 (11)	0.0404 (4)	
H35A	0.5083	0.0771	0.5069	0.049*	
C36	0.61515 (15)	0.08156 (8)	0.40458 (10)	0.0329 (3)	
H36A	0.5469	0.0922	0.3653	0.040*	
C37	0.6986 (2)	0.32495 (8)	0.32085 (12)	0.0444 (4)	
H37A	0.6635	0.3422	0.2671	0.067*	
H37B	0.7906	0.3314	0.3255	0.067*	
H37C	0.6601	0.3460	0.3679	0.067*	
C38	0.73396 (19)	0.23176 (9)	0.43020 (10)	0.0417 (4)	
H38A	0.6910	0.2588	0.4694	0.063*	
H38B	0.8243	0.2424	0.4318	0.063*	
H38C	0.7242	0.1881	0.4469	0.063*	
C39	0.51716 (16)	0.26409 (8)	0.12418 (12)	0.0420 (4)	
H39A	0.5786	0.2918	0.0993	0.063*	
H39B	0.4946	0.2810	0.1787	0.063*	
H39C	0.4408	0.2610	0.0861	0.063*	
C40	0.44968 (15)	0.15096 (9)	0.17929 (12)	0.0426 (4)	
H40A	0.3759	0.1580	0.1399	0.064*	
H40B	0.4332	0.1686	0.2346	0.064*	
H40C	0.4653	0.1060	0.1851	0.064*	
Co1	0.745804 (16)	0.186494 (8)	0.230342 (11)	0.01983 (5)	
O1	0.52521 (11)	0.24091 (7)	0.33284 (8)	0.0494 (3)	
O2	0.59499 (11)	0.16332 (6)	0.05273 (7)	0.0386 (3)	
P1	0.88875 (3)	0.227945 (16)	0.15380 (2)	0.01951 (7)	
P2	0.76289 (3)	0.089222 (16)	0.26405 (2)	0.02134 (8)	
S1	0.66484 (3)	0.242824 (18)	0.32462 (2)	0.02783 (8)	
S2	0.58576 (3)	0.187931 (18)	0.13968 (2)	0.02729 (8)	
H1	0.8514 (17)	0.1820 (8)	0.2884 (11)	0.035 (5)*	

### Atomic displacement parameters (Å^2^)

**Table:**

	*U*^11^	*U*^22^	*U*^33^	*U*^12^	*U*^13^	*U*^23^
C1	0.0224 (6)	0.0223 (7)	0.0240 (6)	−0.0013 (5)	0.0017 (5)	0.0019 (5)
C2	0.0411 (8)	0.0257 (7)	0.0238 (7)	0.0012 (6)	−0.0018 (6)	−0.0012 (6)
C3	0.0641 (11)	0.0329 (9)	0.0259 (7)	−0.0003 (8)	−0.0092 (7)	0.0024 (6)
C4	0.0504 (10)	0.0278 (8)	0.0402 (9)	0.0013 (7)	−0.0127 (7)	0.0079 (7)
C5	0.0479 (10)	0.0264 (8)	0.0403 (9)	0.0079 (7)	0.0031 (7)	0.0019 (7)
C6	0.0429 (9)	0.0286 (8)	0.0273 (7)	0.0058 (6)	0.0052 (6)	0.0000 (6)
C7	0.0208 (6)	0.0200 (6)	0.0255 (6)	0.0012 (5)	0.0007 (5)	−0.0022 (5)
C8	0.0268 (7)	0.0240 (7)	0.0264 (7)	−0.0020 (5)	0.0000 (5)	0.0014 (5)
C9	0.0338 (8)	0.0287 (8)	0.0311 (7)	−0.0007 (6)	−0.0079 (6)	0.0005 (6)
C10	0.0261 (7)	0.0296 (8)	0.0474 (9)	−0.0040 (6)	−0.0098 (6)	0.0003 (7)
C11	0.0246 (7)	0.0279 (8)	0.0451 (9)	−0.0040 (6)	0.0044 (6)	0.0013 (6)
C12	0.0268 (7)	0.0263 (7)	0.0285 (7)	−0.0013 (6)	0.0041 (5)	0.0000 (6)
C13	0.0277 (7)	0.0206 (6)	0.0207 (6)	0.0002 (5)	0.0051 (5)	0.0011 (5)
C14	0.0287 (7)	0.0288 (8)	0.0295 (7)	0.0029 (6)	0.0031 (6)	−0.0024 (6)
C15	0.0328 (8)	0.0348 (9)	0.0379 (8)	0.0091 (6)	0.0083 (6)	−0.0022 (7)
C16	0.0464 (9)	0.0236 (7)	0.0322 (8)	0.0047 (6)	0.0136 (7)	−0.0025 (6)
C17	0.0454 (9)	0.0255 (8)	0.0275 (7)	−0.0054 (6)	0.0052 (6)	−0.0037 (6)
C18	0.0302 (7)	0.0250 (7)	0.0260 (7)	−0.0018 (6)	0.0047 (5)	−0.0008 (6)
C19	0.0265 (7)	0.0261 (7)	0.0223 (6)	0.0034 (5)	0.0024 (5)	0.0006 (5)
C20	0.0278 (7)	0.0292 (8)	0.0337 (8)	0.0023 (6)	0.0012 (6)	0.0042 (6)
C21	0.0253 (7)	0.0457 (10)	0.0385 (8)	0.0025 (7)	0.0015 (6)	0.0067 (7)
C22	0.0328 (8)	0.0494 (10)	0.0325 (8)	0.0174 (7)	0.0018 (6)	0.0003 (7)
C23	0.0428 (9)	0.0337 (9)	0.0443 (9)	0.0143 (7)	−0.0029 (7)	−0.0081 (7)
C24	0.0321 (8)	0.0277 (8)	0.0406 (8)	0.0038 (6)	−0.0007 (6)	−0.0051 (6)
C25	0.0250 (6)	0.0204 (7)	0.0306 (7)	−0.0001 (5)	0.0004 (5)	−0.0010 (5)
C26	0.0309 (7)	0.0274 (8)	0.0301 (7)	0.0001 (6)	0.0017 (6)	−0.0010 (6)
C27	0.0368 (8)	0.0326 (8)	0.0331 (8)	0.0043 (6)	−0.0039 (6)	−0.0061 (6)
C28	0.0367 (8)	0.0307 (8)	0.0485 (10)	−0.0040 (7)	−0.0100 (7)	−0.0081 (7)
C29	0.0358 (8)	0.0292 (8)	0.0502 (10)	−0.0096 (7)	−0.0002 (7)	0.0005 (7)
C30	0.0317 (7)	0.0280 (8)	0.0343 (8)	−0.0053 (6)	0.0022 (6)	0.0020 (6)
C31	0.0322 (7)	0.0191 (7)	0.0252 (6)	−0.0012 (5)	0.0036 (5)	0.0002 (5)
C32	0.0365 (8)	0.0297 (8)	0.0285 (7)	0.0002 (6)	0.0012 (6)	−0.0015 (6)
C33	0.0547 (10)	0.0365 (9)	0.0266 (7)	0.0011 (8)	−0.0033 (7)	0.0005 (7)
C34	0.0655 (12)	0.0356 (9)	0.0258 (7)	−0.0020 (8)	0.0118 (7)	−0.0004 (7)
C35	0.0469 (10)	0.0380 (9)	0.0383 (9)	0.0000 (8)	0.0182 (7)	−0.0003 (7)
C36	0.0347 (8)	0.0332 (8)	0.0316 (8)	0.0014 (6)	0.0072 (6)	0.0011 (6)
C37	0.0690 (12)	0.0262 (8)	0.0396 (9)	0.0034 (8)	0.0161 (8)	−0.0043 (7)
C38	0.0618 (11)	0.0378 (9)	0.0260 (7)	0.0032 (8)	0.0071 (7)	−0.0050 (7)
C39	0.0346 (8)	0.0396 (10)	0.0505 (10)	0.0120 (7)	−0.0082 (7)	−0.0003 (8)
C40	0.0218 (7)	0.0516 (11)	0.0540 (10)	−0.0054 (7)	−0.0001 (7)	−0.0013 (8)
Co1	0.01897 (9)	0.02004 (10)	0.02051 (9)	0.00010 (7)	0.00147 (6)	−0.00072 (7)
O1	0.0312 (6)	0.0681 (9)	0.0503 (7)	0.0022 (6)	0.0149 (5)	−0.0158 (6)
O2	0.0350 (6)	0.0497 (7)	0.0301 (6)	0.0051 (5)	−0.0059 (4)	−0.0082 (5)
P1	0.02040 (15)	0.01970 (16)	0.01851 (15)	−0.00003 (12)	0.00179 (12)	−0.00071 (12)
P2	0.02156 (16)	0.01971 (17)	0.02289 (16)	−0.00094 (13)	0.00256 (13)	−0.00041 (13)
S1	0.02933 (17)	0.02839 (19)	0.02654 (17)	0.00214 (14)	0.00784 (13)	−0.00359 (14)
S2	0.02106 (16)	0.03158 (19)	0.02882 (17)	0.00215 (13)	−0.00186 (13)	−0.00197 (14)

### Geometric parameters (Å, °)

**Table:**

C1—C2	1.3873 (19)	C25—C26	1.394 (2)
C1—C6	1.392 (2)	C25—C30	1.395 (2)
C1—P1	1.8489 (14)	C25—P2	1.8464 (14)
C2—C3	1.3850 (16)	C26—C27	1.385 (2)
C2—H2A	0.9500	C26—H26A	0.9500
C3—C4	1.3808 (17)	C27—C28	1.381 (2)
C3—H3A	0.9500	C27—H27A	0.9500
C4—C5	1.381 (2)	C28—C29	1.379 (3)
C4—H4A	0.9500	C28—H28A	0.9500
C5—C6	1.384 (2)	C29—C30	1.390 (2)
C5—H5A	0.9500	C29—H29A	0.9500
C6—H6A	0.9500	C30—H30A	0.9500
C7—C8	1.3879 (19)	C31—C36	1.392 (2)
C7—C12	1.3963 (19)	C31—C32	1.392 (2)
C7—P1	1.8477 (13)	C31—P2	1.8431 (14)
C8—C9	1.389 (2)	C32—C33	1.3853 (16)
C8—H8A	0.9500	C32—H32A	0.9500
C9—C10	1.378 (2)	C33—C34	1.3811 (18)
C9—H9A	0.9500	C33—H33A	0.9500
C10—C11	1.382 (2)	C34—C35	1.382 (3)
C10—H10A	0.9500	C34—H34A	0.9500
C11—C12	1.387 (2)	C35—C36	1.383 (2)
C11—H11A	0.9500	C35—H35A	0.9500
C12—H12A	0.9500	C36—H36A	0.9500
C13—C14	1.389 (2)	C37—S1	1.7967 (17)
C13—C18	1.397 (2)	C37—H37A	0.9800
C13—P1	1.8449 (14)	C37—H37B	0.9800
C14—C15	1.391 (2)	C37—H37C	0.9800
C14—H14A	0.9500	C38—S1	1.7995 (17)
C15—C16	1.378 (2)	C38—H38A	0.9800
C15—H15A	0.9500	C38—H38B	0.9800
C16—C17	1.381 (2)	C38—H38C	0.9800
C16—H16A	0.9500	C39—S2	1.7958 (17)
C17—C18	1.384 (2)	C39—H39A	0.9800
C17—H17A	0.9500	C39—H39B	0.9800
C18—H18A	0.9500	C39—H39C	0.9800
C19—C20	1.387 (2)	C40—S2	1.7881 (17)
C19—C24	1.391 (2)	C40—H40A	0.9800
C19—P2	1.8559 (14)	C40—H40B	0.9800
C20—C21	1.3873 (16)	C40—H40C	0.9800
C20—H20A	0.9500	Co1—S1	2.1387 (4)
C21—C22	1.3831 (17)	Co1—S2	2.1507 (4)
C21—H21A	0.9500	Co1—P2	2.1559 (4)
C22—C23	1.381 (3)	Co1—P1	2.1823 (4)
C22—H22A	0.9500	Co1—H1	1.405 (18)
C23—C24	1.385 (2)	O1—S1	1.4891 (12)
C23—H23A	0.9500	O2—S2	1.4837 (11)
C24—H24A	0.9500		
			
C2—C1—C6	118.17 (13)	C27—C28—H28A	120.3
C2—C1—P1	124.47 (11)	C28—C29—C30	120.67 (15)
C6—C1—P1	117.30 (10)	C28—C29—H29A	119.7
C3—C2—C1	120.40 (14)	C30—C29—H29A	119.7
C3—C2—H2A	119.8	C29—C30—C25	120.36 (15)
C1—C2—H2A	119.8	C29—C30—H30A	119.8
C4—C3—C2	120.72 (15)	C25—C30—H30A	119.8
C4—C3—H3A	119.6	C36—C31—C32	118.06 (13)
C2—C3—H3A	119.6	C36—C31—P2	119.27 (11)
C3—C4—C5	119.47 (14)	C32—C31—P2	122.42 (11)
C3—C4—H4A	120.3	C33—C32—C31	120.86 (14)
C5—C4—H4A	120.3	C33—C32—H32A	119.6
C4—C5—C6	119.69 (15)	C31—C32—H32A	119.6
C4—C5—H5A	120.2	C34—C33—C32	120.19 (16)
C6—C5—H5A	120.2	C34—C33—H33A	119.9
C5—C6—C1	121.37 (14)	C32—C33—H33A	119.9
C5—C6—H6A	119.3	C33—C34—C35	119.70 (14)
C1—C6—H6A	119.3	C33—C34—H34A	120.1
C8—C7—C12	118.10 (13)	C35—C34—H34A	120.1
C8—C7—P1	121.25 (10)	C34—C35—C36	119.99 (16)
C12—C7—P1	120.65 (11)	C34—C35—H35A	120.0
C7—C8—C9	121.37 (13)	C36—C35—H35A	120.0
C7—C8—H8A	119.3	C35—C36—C31	121.12 (15)
C9—C8—H8A	119.3	C35—C36—H36A	119.4
C10—C9—C8	119.69 (14)	C31—C36—H36A	119.4
C10—C9—H9A	120.2	S1—C37—H37A	109.5
C8—C9—H9A	120.2	S1—C37—H37B	109.5
C9—C10—C11	119.89 (14)	H37A—C37—H37B	109.5
C9—C10—H10A	120.1	S1—C37—H37C	109.5
C11—C10—H10A	120.1	H37A—C37—H37C	109.5
C10—C11—C12	120.33 (14)	H37B—C37—H37C	109.5
C10—C11—H11A	119.8	S1—C38—H38A	109.5
C12—C11—H11A	119.8	S1—C38—H38B	109.5
C11—C12—C7	120.52 (14)	H38A—C38—H38B	109.5
C11—C12—H12A	119.7	S1—C38—H38C	109.5
C7—C12—H12A	119.7	H38A—C38—H38C	109.5
C14—C13—C18	118.17 (13)	H38B—C38—H38C	109.5
C14—C13—P1	124.44 (11)	S2—C39—H39A	109.5
C18—C13—P1	117.14 (10)	S2—C39—H39B	109.5
C13—C14—C15	120.67 (14)	H39A—C39—H39B	109.5
C13—C14—H14A	119.7	S2—C39—H39C	109.5
C15—C14—H14A	119.7	H39A—C39—H39C	109.5
C16—C15—C14	120.47 (15)	H39B—C39—H39C	109.5
C16—C15—H15A	119.8	S2—C40—H40A	109.5
C14—C15—H15A	119.8	S2—C40—H40B	109.5
C15—C16—C17	119.50 (14)	H40A—C40—H40B	109.5
C15—C16—H16A	120.2	S2—C40—H40C	109.5
C17—C16—H16A	120.2	H40A—C40—H40C	109.5
C16—C17—C18	120.28 (15)	H40B—C40—H40C	109.5
C16—C17—H17A	119.9	S1—Co1—S2	97.289 (15)
C18—C17—H17A	119.9	S1—Co1—P2	113.785 (15)
C17—C18—C13	120.90 (14)	S2—Co1—P2	103.285 (15)
C17—C18—H18A	119.5	S1—Co1—P1	118.569 (16)
C13—C18—H18A	119.5	S2—Co1—P1	99.515 (15)
C20—C19—C24	118.09 (13)	P2—Co1—P1	118.725 (15)
C20—C19—P2	120.81 (11)	S1—Co1—H1	85.2 (7)
C24—C19—P2	121.09 (11)	S2—Co1—H1	176.8 (7)
C19—C20—C21	120.89 (14)	P2—Co1—H1	73.8 (7)
C19—C20—H20A	119.6	P1—Co1—H1	80.9 (7)
C21—C20—H20A	119.6	C13—P1—C7	102.20 (6)
C22—C21—C20	120.41 (15)	C13—P1—C1	102.05 (6)
C22—C21—H21A	119.8	C7—P1—C1	98.42 (6)
C20—C21—H21A	119.8	C13—P1—Co1	113.87 (4)
C23—C22—C21	119.20 (14)	C7—P1—Co1	117.52 (4)
C23—C22—H22A	120.4	C1—P1—Co1	119.93 (4)
C21—C22—H22A	120.4	C31—P2—C25	102.13 (7)
C22—C23—C24	120.34 (16)	C31—P2—C19	99.69 (6)
C22—C23—H23A	119.8	C25—P2—C19	97.94 (6)
C24—C23—H23A	119.8	C31—P2—Co1	112.92 (5)
C23—C24—C19	121.05 (15)	C25—P2—Co1	122.09 (5)
C23—C24—H24A	119.5	C19—P2—Co1	118.51 (5)
C19—C24—H24A	119.5	O1—S1—C37	103.26 (9)
C26—C25—C30	118.31 (13)	O1—S1—C38	105.24 (8)
C26—C25—P2	115.77 (11)	C37—S1—C38	95.15 (9)
C30—C25—P2	125.83 (11)	O1—S1—Co1	119.07 (5)
C27—C26—C25	120.88 (14)	C37—S1—Co1	116.15 (6)
C27—C26—H26A	119.6	C38—S1—Co1	114.71 (6)
C25—C26—H26A	119.6	O2—S2—C40	105.56 (8)
C28—C27—C26	120.35 (15)	O2—S2—C39	104.16 (8)
C28—C27—H27A	119.8	C40—S2—C39	97.12 (9)
C26—C27—H27A	119.8	O2—S2—Co1	121.43 (5)
C29—C28—C27	119.41 (15)	C40—S2—Co1	112.03 (6)
C29—C28—H28A	120.3	C39—S2—Co1	113.48 (6)
			
C6—C1—C2—C3	−3.8 (2)	C2—C1—P1—C13	15.96 (14)
P1—C1—C2—C3	173.25 (13)	C6—C1—P1—C13	−166.97 (12)
C1—C2—C3—C4	0.4 (3)	C2—C1—P1—C7	120.44 (13)
C2—C3—C4—C5	2.8 (3)	C6—C1—P1—C7	−62.49 (12)
C3—C4—C5—C6	−2.5 (3)	C2—C1—P1—Co1	−110.94 (12)
C4—C5—C6—C1	−1.0 (3)	C6—C1—P1—Co1	66.13 (12)
C2—C1—C6—C5	4.1 (2)	S1—Co1—P1—C13	171.96 (5)
P1—C1—C6—C5	−173.17 (13)	S2—Co1—P1—C13	−84.28 (5)
C12—C7—C8—C9	−1.6 (2)	P2—Co1—P1—C13	26.67 (5)
P1—C7—C8—C9	178.77 (11)	S1—Co1—P1—C7	52.55 (5)
C7—C8—C9—C10	−1.4 (2)	S2—Co1—P1—C7	156.30 (5)
C8—C9—C10—C11	2.9 (2)	P2—Co1—P1—C7	−92.75 (5)
C9—C10—C11—C12	−1.2 (2)	S1—Co1—P1—C1	−66.82 (5)
C10—C11—C12—C7	−1.8 (2)	S2—Co1—P1—C1	36.93 (5)
C8—C7—C12—C11	3.2 (2)	P2—Co1—P1—C1	147.88 (5)
P1—C7—C12—C11	−177.15 (11)	C36—C31—P2—C25	−63.39 (13)
C18—C13—C14—C15	0.2 (2)	C32—C31—P2—C25	122.43 (13)
P1—C13—C14—C15	174.30 (12)	C36—C31—P2—C19	−163.76 (12)
C13—C14—C15—C16	0.6 (2)	C32—C31—P2—C19	22.06 (14)
C14—C15—C16—C17	−0.5 (2)	C36—C31—P2—Co1	69.52 (13)
C15—C16—C17—C18	−0.4 (2)	C32—C31—P2—Co1	−104.66 (12)
C16—C17—C18—C13	1.2 (2)	C26—C25—P2—C31	−175.08 (11)
C14—C13—C18—C17	−1.1 (2)	C30—C25—P2—C31	1.57 (14)
P1—C13—C18—C17	−175.62 (11)	C26—C25—P2—C19	−73.32 (12)
C24—C19—C20—C21	1.2 (2)	C30—C25—P2—C19	103.32 (13)
P2—C19—C20—C21	179.90 (12)	C26—C25—P2—Co1	57.69 (12)
C19—C20—C21—C22	0.0 (2)	C30—C25—P2—Co1	−125.66 (12)
C20—C21—C22—C23	−1.1 (2)	C20—C19—P2—C31	−97.04 (12)
C21—C22—C23—C24	1.0 (3)	C24—C19—P2—C31	81.58 (13)
C22—C23—C24—C19	0.2 (3)	C20—C19—P2—C25	159.12 (12)
C20—C19—C24—C23	−1.4 (2)	C24—C19—P2—C25	−22.25 (13)
P2—C19—C24—C23	179.98 (13)	C20—C19—P2—Co1	25.80 (13)
C30—C25—C26—C27	−0.5 (2)	C24—C19—P2—Co1	−155.58 (11)
P2—C25—C26—C27	176.40 (12)	S1—Co1—P2—C31	−12.08 (5)
C25—C26—C27—C28	1.6 (2)	S2—Co1—P2—C31	−116.35 (5)
C26—C27—C28—C29	−1.4 (2)	P1—Co1—P2—C31	134.80 (5)
C27—C28—C29—C30	0.1 (3)	S1—Co1—P2—C25	110.23 (6)
C28—C29—C30—C25	1.0 (3)	S2—Co1—P2—C25	5.96 (6)
C26—C25—C30—C29	−0.8 (2)	P1—Co1—P2—C25	−102.89 (6)
P2—C25—C30—C29	−177.34 (12)	S1—Co1—P2—C19	−128.03 (5)
C36—C31—C32—C33	1.1 (2)	S2—Co1—P2—C19	127.70 (5)
P2—C31—C32—C33	175.39 (12)	P1—Co1—P2—C19	18.85 (5)
C31—C32—C33—C34	1.4 (3)	S2—Co1—S1—O1	30.74 (7)
C32—C33—C34—C35	−2.1 (3)	P2—Co1—S1—O1	−77.29 (7)
C33—C34—C35—C36	0.4 (3)	P1—Co1—S1—O1	135.77 (7)
C34—C35—C36—C31	2.2 (3)	S2—Co1—S1—C37	−93.69 (8)
C32—C31—C36—C35	−2.9 (2)	P2—Co1—S1—C37	158.28 (8)
P2—C31—C36—C35	−177.33 (13)	P1—Co1—S1—C37	11.35 (8)
C14—C13—P1—C7	10.10 (14)	S2—Co1—S1—C38	156.68 (7)
C18—C13—P1—C7	−175.78 (11)	P2—Co1—S1—C38	48.64 (7)
C14—C13—P1—C1	111.59 (13)	P1—Co1—S1—C38	−98.29 (7)
C18—C13—P1—C1	−74.28 (12)	S1—Co1—S2—O2	170.37 (6)
C14—C13—P1—Co1	−117.68 (12)	P2—Co1—S2—O2	−73.02 (7)
C18—C13—P1—Co1	56.44 (12)	P1—Co1—S2—O2	49.69 (7)
C8—C7—P1—C13	−120.84 (12)	S1—Co1—S2—C40	−63.72 (7)
C12—C7—P1—C13	59.52 (12)	P2—Co1—S2—C40	52.90 (7)
C8—C7—P1—C1	134.81 (12)	P1—Co1—S2—C40	175.60 (7)
C12—C7—P1—C1	−44.83 (12)	S1—Co1—S2—C39	45.04 (7)
C8—C7—P1—Co1	4.58 (13)	P2—Co1—S2—C39	161.66 (7)
C12—C7—P1—Co1	−175.07 (10)	P1—Co1—S2—C39	−75.64 (7)
